# Sensitivity and specificity of the abbreviated profile of hearing aid benefit (APHAB)

**DOI:** 10.1007/s00405-017-4680-y

**Published:** 2017-07-29

**Authors:** Jan Löhler, F. Gräbner, B. Wollenberg, P. Schlattmann, R. Schönweiler

**Affiliations:** 1 Scientific Institute for Applied ENT-Research of the German Professional Association of ENT-Surgeons, Bad Bramstedt, Germany; 2 Wissenschaftliches Institut für angewandte HNO-Heilkunde (WIAHNO) des Deutschen Berufsverbandes der HNO-Ärzte e. V., Maienbeeck 1, 24576 Bad Bramstedt, Germany; 3 Department of ENT-Surgery, University Hospital Schleswig-Holstein, Campus Luebeck, Luebeck, Germany; 4 Klinik für HNO-Heilkunde, Universitätsklinikum Schleswig-Holstein, Campus Lübeck, Luebeck, Germany; 5 Institute for Medical Statistics, Informatics and Documentations, University Hospital Jena, Jena, Germany; 6 Section of Phoniatrics and Pedaudiology in the Department of ENT-Surgery, University Hospital of Schleswig-Holstein, Campus Luebeck, Luebeck, Germany; 7 Sektion für Phoniatrie und Pädaudiologie in der Klinik für Hals-Nasen-Ohrenheilkunde, Universitätsklinikum Schleswig-Holstein, Campus Lübeck, Luebeck, Germany

**Keywords:** Abbreviated profile of hearing aid benefit, APHAB, APHAB_u_, Inventory, Questionnaire, Hearing loss, Hearing aid fitting, Sensitivity, Specificity, Diagnostic value

## Abstract

Subjective hearing loss in hearing-impaired patients can be assessed by inventory questionnaires. The abbreviated profile of hearing aid benefit (APHAB) measures subjective hearing loss in four typical hearing situations (subscales). It is used to fit hearing aids in patients with statutory insurance in Germany. In addition, the unaided APHAB (APHAB_u_) can be used as a primary diagnostic instrument in audiology. There are no published data regarding the sensitivity and specificity of the unaided APHAB_u_. Therefore, we investigated these parameters for detecting hearing loss of at least 25 dB at any frequency between 0.5 and 8.0 kHz. We used the APHAB_u_ to determine hearing loss in 245 subjects aged 50 years and older without any reported disease of the ears. Due to incomplete answering of the APHAB form, 55 subjects have been excluded. We also measured the pure-tone thresholds by air conduction for all octave frequencies between 0.5 and 8 kHz. Receiver operating characteristic (ROC) curves and the Youden Index were used to determine the diagnostic value of the APHAB_u_, particularly sensitivity and specificity, in three different ways: (1) separately for ease of communication (EC_u_), background noise (BN_u_), and hearing with reverberation (RV_u_) subscales; (2) with the mean value of EC_u_, BN_u_, and RV_u_; and (3) with a logistic regression model. The area under the ROC curve was lower for BN only (0.83) and nearly equal for all other methods (0.87–0.89). Depending on how we performed the analyses, the sensitivity of the APHAB_u_ was 0.70–0.84 (single subscales), 0.76 (mean value of EC_u_, BN_u_, and RV_u_), or 0.85 (logistic regression model). The specificity was 0.79–0.95. The use of single APHAB_u_ subscales for determining the sensitivity and specificity of the APHAB_u_ due to confusing results. In comparison, the use of the mean value of EC_u_, BN_u_, and RV_u_ and the use of the logistic regression model due to equal values in the ROC curves but a higher sensitivity in the logistic regression model. Therefore, we would recommend the last method for determining the sensitivity and specificity of the APHAB_u_.

## Introduction

Grades of hearing loss are objectively measured by pure-tone and speech audiometry while self-reporting questionnaires can be used to measure subjective hearing impairment. Recently, the abbreviated profile of hearing aid benefit (APHAB), developed by Cox and Alexander [1, 2], has become an important audiological tool in Germany [3, 4], and it is currently the most commonly used diagnostic inventory for patients with statutory insurance in this country. There are no discernible differences between the original United States APHAB and the German adaptation [5, 6].

The APHAB comprises 24 single questions divided into four subscales that measure hearing loss in everyday hearing situations. The ease of communication (EC) scale examines basic hearing situations without ambient noise in a quiet environment, the background noise (BN) scale examines hearing situations with background noise, the reverberation (RV) scale investigates hearing situations in large spaces with echoes, and the aversiveness (AV) scale measures the perception of loud sound events.

Löhler et al. [7] recently demonstrated that there is an association between hearing loss in the 0.5–4.0 kHz octave frequencies and unaided APHAB scores (APHAB_u_) in the EC and RV subscales. In contrast, they did not find any association between individual hearing loss typified to standard audiograms introduced by [8] and APHAB_u_ scores in any subscale [9]. Moreover, they found that the majority of respondents answered most questions; those few questions (Question numbers 11, 18, and 21) that were answered less frequently may have been less relevant to everyday situations [10]. Finally, Löhler et al. [11] investigated associations between APHAB_u_ scores and hearing loss at all octave frequencies between 0.5 and 8 kHz. Moreover, they investigated all of the subscales in 5% steps and 5 dB level steps were investigated [11]. Löhler et al.’s multiple investigations demonstrated a relatively high association between hearing loss and APHAB_u_ scores for the EC and RV subscales, but not for the BN subscale. As in speech audiometry, this is probably due to the ability of individuals to learn how to compensate for BN. Hearing with RV is rarer than problems with BN; thus, there are fewer opportunities for individuals to experience RV and develop ways to compensate for it. Moreover, hearing problems in normal, low-noise conditions (EC) generally affect only individuals with severe hearing loss, with, again, fewer opportunities to learn how to compensate. The AV subscale score does appear to be negatively associated with hearing loss [7, 10]. The three subscales EC, BN, and RV assess understanding in different situations. In general, increasing levels of hearing loss are associated with increasing scores in the specific subscale and opposite, as has been demonstrated [7, 9]. In contrast, the AV subscale, detecting how noisy situations were misperceived (respectively, the aversiveness of sounds), is characterized by decreasing APHAB_u_ values correlated to increasing dB values of hearing loss [7].

To the best of our knowledge, no published studies have addressed the sensitivity and specificity of the APHAB for detecting a defined hearing loss. This lack of data is typical of most inventories [12]. Because of the important role that the APHAB plays in healthcare in Germany, the aim of the present study was to measure the sensitivity and specificity of the APHAB for detecting a hearing loss of at least 25 dB in one or more of the octave frequencies between 0.5 and 8.0 kHz by the following three methods: (1) separate calculations for the EC_u_, BN_u_, and RV_u_ subscales; (2) calculation of the mean of the EC_u_, BN_u_, and RV_u_ subscales; and (3) a logistic regression model for the EC_u_, BN_u_, and RV_u_ subscales. Due to the mentioned effect of the opposite character of the AV subscale (measuring the aversiveness of loud situations), it is not rational to include the AV subscale for determining the sensitivity and specificity of the APHAB_u_. The high variance of individual compensating effects will limit the use of BN subscale for the detection of sensitivity and specificity as well. Therefore, it may be of benefit to focus on the EC and RV subscales within our investigation.

With the values of specificity and sensitivity, the last missing main characteristic of the APHAB will be described. Together with the known attributes [7, 9–11] the results of individual APHAB scores could be well interpreted on the background of anamnesis and the data of pure-tone and speech audiometry to evaluate a specific hearing loss.

## Methods

In Germany, an APHAB database has been established several years ago [13]. Between 1 May 2016 and 30 June 2016, we administered the APHAB_u_ to 245 subjects aged 50 years and older who had no actual or reported disease of the ears or hearing impairment. Thus, we used the APHAB as a primary diagnostic tool for hearing loss. In addition, we measured and recorded the pure-tone thresholds of the participants at all octave frequencies between 0.5 and 8.0 kHz by air conduction. The database did not include a record of patients who had a difference in hearing loss of >60 dB in comparisons of air conduction for both ears at frequencies at 0.5, 1.0, and 2.0 kHz, based on the three-frequency table [5, 6]. We excluded these patients to avoid the influence of compensatory effects in cases of severe hearing loss asymmetry. In addition, this should make our results comparable to other APHAB investigations which used the same condition [5–7, 9–11]. We collected data both via an online questionnaire method and from traditional paper-and-pencil questionnaires and later database entering via internet-based access. All data were stored on a central server. The subjects’ participation in data storage was voluntary. The Ethics Commission of the Schleswig-Holstein Medical Association and the state data protection officer approved the research methods.

We used receiver operating characteristic (ROC) curves [14, 15] to evaluate the sensitivity and specificity of the APHAB_u_ to detect a hearing loss of 25 dB in at least one of the octave frequencies between 0.5 and 8.0 kHz in any ear. Using 5 dB steps in pure-tone thresholds, this will lead to the same results as using a cutoff value for hearing loss of more than 20 dB. Logistic regression was used to construct the ROC curves. We determined the diagnostic value of the APHAB_u_ by the area under the ROC curve (AUC) with 95% confidence intervals. The threshold of the APHAB_u_ was identified with the Youden Index [16] and calculated by sensitivity + specificity − 1.

In accordance with the aim of our study, we analyzed all of the data to determine the sensitivity and specificity of the APHAB_u_ by means of the following three methods:

1. We determined the optimal cutoff values for detecting hearing loss by considering EC_u_, BN_u_, and RV_u_ individually.
2. We determined the optimal cutoff value using the arithmetic mean value of the unaided EC, BN, and RV subscales:
  1 $$\bar{x} = \frac{{{\text{EC}}_{\text{u}} {\text{ + BN}}_{\text{u}} {\text{ + RV}}_{\text{u}} }}{3}.$$
3. We determined the probability (cutoff) for the unaided EC and RV subscales with a logistic transforming regression analysis mode using a logistic regression model with random effects [17]. The following equation shows the fixed effects for the model at hand. Here *p* denotes the probability of hearing loss in any frequency ranging from 0.5 to 8.0 kHz:
  2a $$\ln \left( {\frac{p}{1 - p}} \right) = a + b{\text{EC}}_{\text{u}} { + }c{\text{RV}}_{\text{u}} .$$

The hearing loss result of the audiogram was the dependent variable and the APHAB_u_ scores were the independent variables. Gender and site of hearing loss were additional independent variables. The choice of a random effect model serves two purposes. One, we are able to model variability between patients and second we are able to take the paired data structure into account (left vs. right ear). Calculations were performed with SAS software version 9.4, PROC GLIMMIX (Table 1).

**Table 1:** Three-frequency table to define the degree of hearing impairment Adapted from [5, 6]

	Hearing loss at 2.0 kHz				
	<20 dB	20–35 dB	40–55 dB	60–80 dB	>80 dB
Total hearing loss at 0.5 and 1.0 kHz					
0–35 dB	None	Slight	Moderate	Moderate–profound	Profound
40–75 dB	Slight	Slight	Moderate	Moderate–profound	Profound
80–115 dB	Moderate	Moderate	Moderate	Moderate–profound	Profound
120–160 dB	Moderate–profound	Moderate–profound	Moderate–profound	Moderate–profound	Profound
>160 dB	Profound	Profound	Profound	Profound	Profound

Findings from the sound audiogram of the inferior ear measured in 5-dB steps. Subjects with a difference of >60 dB of hearing loss between the left and right ears were initially excluded from the database

## Results

### General characteristics of the study participants

The average age of all 245 participants was 58.0 years and the median age was 59.0 years. One hundred and thirty-three of the subjects were men (54.3%, average age 58.7 ± 12.4 years) while one hundred and twelve were women (45.7%, average age 57.0 ± 12.5 years). Forty-three of the participants (17.6%) had normal thresholds (maximum hearing loss of 20 dB in one or more octave frequencies between 0.5 and 8.0 kHz). Fifty-five subjects (22.4%) did not answer all of the APHAB questions; this left 190 full data sets for analysis in this study. APHAB_u_ values were independent of ear side and gender. Table 2 demonstrates the mean APHAB_u_ values for all subscales and belonging standard deviations. Table 3 shows the mean hearing losses and standard deviations for all frequencies, groups of hearing loss (without and with hearing loss), and the side of the ears.

**Table 2:** Mean APHAB_u_ values and standard deviation for each subscale for all groups

APHAB_u_ subscale	Mean all subjects	Standard deviation	Mean group normal hearing	Standard deviation	Mean group hearing loss	Standard deviation
EC	23.28	24.29	4.98	9.77	27.34	24.68
BN	37.75	25.57	15.72	15.05	42.79	24.82
RV	32.71	25.50	8.73	7.94	38.56	24.88
AV	37.89	25.48	36.00	25.85	38.29	25.45

**Table 3:** Mean hearing loss vs. ear site, frequency, and patient group

Group	Side of ear	frequency (kHz)	Mean hearing loss (dB)	Standard deviation
Normal hearing	Left	0.5	10.73	4.69
		1.0	9.39	5.83
		2.0	11.10	5.76
		4.0	12.93	6.12
		8.0	11.34	6.89
	Right	0.5	10.49	5.34
		1.0	9.76	6.02
		2.0	12.20	5.71
		4.0	12.93	5.47
		8.0	13.54	6.25
With hearing loss	Left	0.5	24.28	13.74
		1.0	26.06	15.60
		2.0	35.66	19.81
		4.0	50.95	21.16
		8.0	55.16	20.93
	Right	0.5	23.39	12.72
		1.0	26.28	14.68
		2.0	34.61	18.88
		4.0	47.57	21.00
		8.0	51.68	21.32

### APHAB_u_ sensitivity and specificity according to the three models

**Fig. 1:**
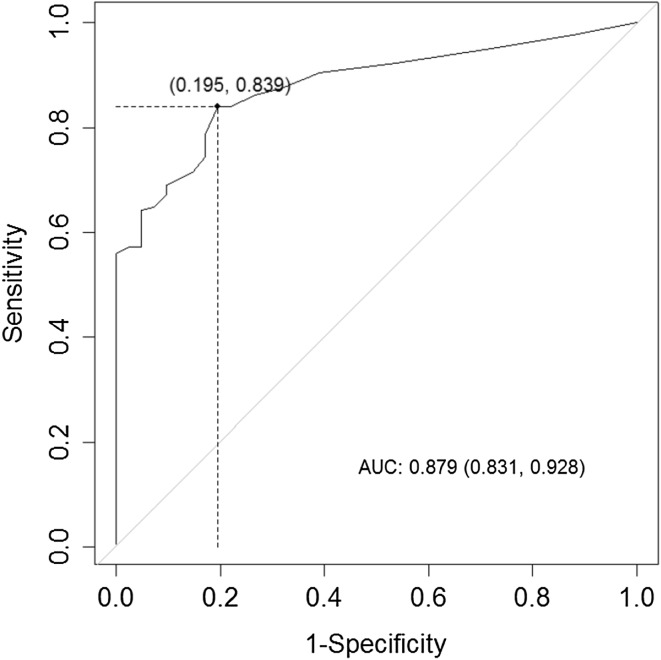
ROC curve for detecting a hearing loss of 25 dB using the RV subscale score

**Fig. 2:**
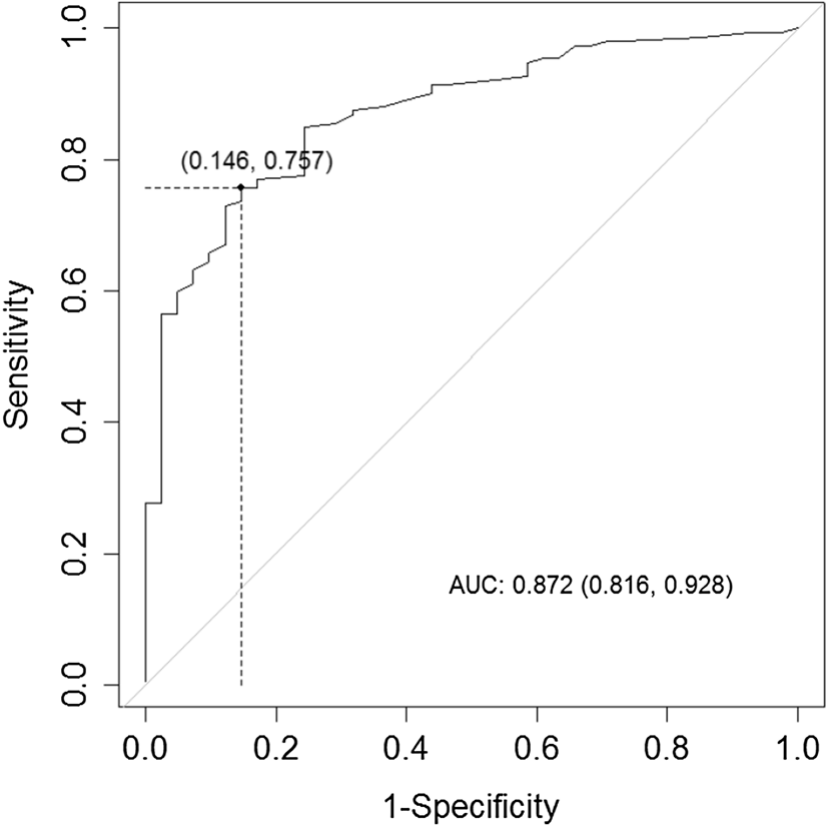
ROC curve for detecting a hearing loss of 25 dB using the mean of the EC, BN, and RV scores

**Fig. 3:**
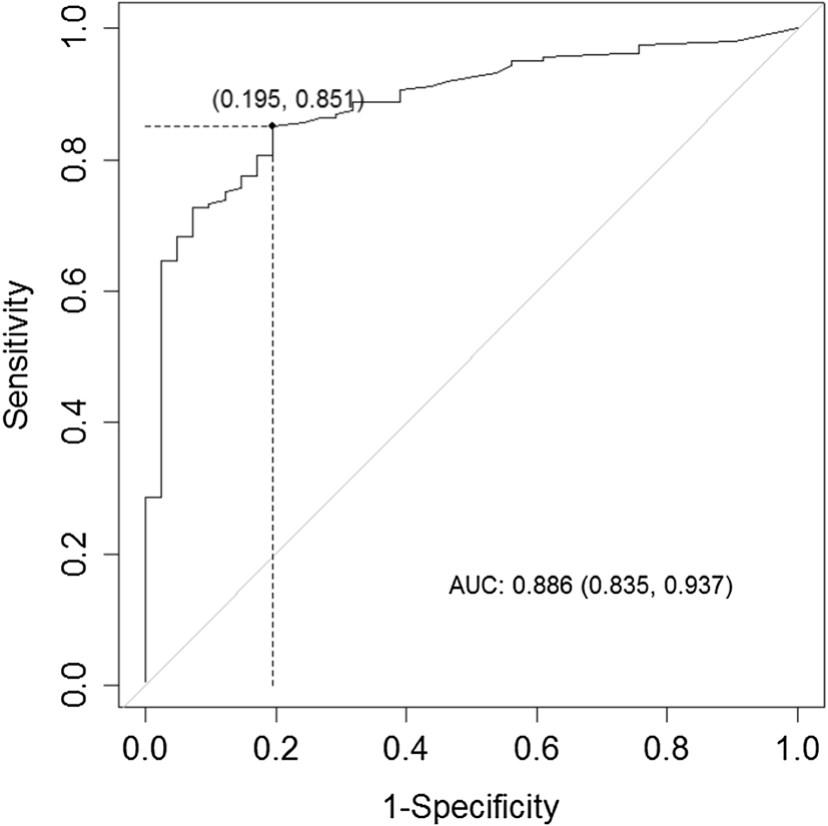
ROC curve for detecting a hearing loss of 25 dB using the logistic regression model

Table 4 contains the results of our Youden Index and ROC curve analyses of the optimal cutoff points for the EC_u_, BN_u_, and RV_u_ scores and the ability of the average score of these subscales (Eq. 1) to detect any hearing loss of ≥25 dB in one or more of the octave frequencies between 0.5 and 8.0 kHz. The ROC curves for RV_u_ only; for the mean value of EC_u_, BN_u_, and RV_u_; and for the logistic regression model are presented in Figs. 1, 2, and 3. The probability (cutoff) determined by the Youden Index and ROC curve for logistic regression (Eq. 2a) was 0.63 (Table 4). We used this value in a multivariate mixed linear model for fixed effects based on the values of the constants *a*, *b*, and *c* in Eq. (2a) (Table 5):

2b $$\ln \left( {\frac{p}{1 - p}} \right) = - 0.82 + 0.06*{\text{EC}}_{\text{u}} + 0.09*{\text{RV}}_{\text{u}} .$$

**Table 4:** Diagnostic value of (1) individual APHAB_u_ subscales; (2) average value of EC_u_, BN_u_, and RV_u_ subscales ($\bar{x}$); and (3) logistic regression model

APHAB_u_ subscale	EC_u_	BN_u_	RV_u_	$\bar{x}$	Logistic model
Cutoff value	0.10	0.23	0.12	0.15	0.63
Area under curve	0.88	0.83	0.88	0.87	0.89
95% CI					
Lower	0.82	0.76	0.83	0.81	0.84
Upper	0.93	0.89	0.93	0.93	0.94
Sensitivity	0.70	0.75	0.84	0.76	0.85
95% CI					
Lower	0.63	0.68	0.77	0.68	0.79
Upper	0.76	0.81	0.89	0.82	0.90
Specificity	0.95	0.79	0.81	0.85	0.81
95% CI					
Lower	0.84	0.64	0.65	0.71	0.65
Upper	0.99	0.90	0.91	0.94	0.91

Cutoff value: APHAB_u_ score for the presented values of sensitivity and specificity

*95% CI* 95% confidence interval

**Table 5:** Values of constants for Eq. (2a) (logistic regression model, see text)

Constant	Value	*p*	95% confidence interval	
			Lower	Upper
a. Intercept	−0.82	0.02	−1.50	−0.14
b. EC	0.06	0.12	−0.01	0.13
c. RV	0.09	<0.01	0.04	0.14

Because of the natural logarithmic scale of the calculated values in the second column of Table 5, the influence of RV_u_ was twice as large as that of EC_u_.

## Discussion

We found that neither the side of the ear nor gender influenced the APHAB_u_ score. This finding is in concordance with those of previously published reports. The mean age of our group of participants was younger than studies that included subjects with are subsequently fitted with hearing aids [7, 9, 10]. According to previous results, the AV subscale is different for all others. Measuring the aversiveness of sounds due to very similar APHAB_u_ scores in both investigated groups (normal hearing and with hearing loss), as demonstrated in Table 2. Using single subscales of APHAB_u_ (EC, BN, and RV) leads to nearly similar cutoff values for EC_u_ and RV_u_, and, by comparison, the cutoff score for BN_u_ was even higher (Table 4). This may be explained by more widespread individual compensation abilities for hearing loss, as shown previously [9, 10]. In addition, the cutoff vaulue for the average model (Eq. 1) due to a value (0.15) which is closer to the values of EC_u_ (0.10) and RV_u_ (0.12) than the BN_u_ value (0.23) by the single use of the subscales. As has been reported, BN subscale scores are not associated with individual hearing loss [7]. Maybe, the lower APHAB_u_ values of the normal hearing group in EC and RV (Table 2) could support this thesis. In addition, the sensitivity using single subscales is resulting in different values around 0.70 and 0.84, whereas the mean subscale (Eq. 1) is 0.76. Although both models are due to values within the confidence intervals, the average model (Eq. 1) may be superior to use of the individual subscales. At least, it is simpler to use one value in sensitivity and specificity than three. This level of sensitivity and specificity is as high as that of other inventories, such as the hearing handicap inventory for the elderly screening version (HHIE-S, [18]) and the Mini-Audio-Test (MAT, [19]). Subjects in the group with false-negative results may ignore their hearing problems, or they may be able to compensate for their hearing impairment. An alternative model is the logistic regression model (Eq. 2b), which uses the constants from Table 5. This model has an even higher level of sensitivity, but its specificity is slightly lower than that of the average model (Eq. 1).

It might be surprising that a hearing loss of 25 dB has an influence on APHAB_u_ scores. In fact, including 8.0 kHz might be very strict and not used in MAT [19], and increases at least the number of healthy or sick ears. But our findings confirm previous results [7, 10]. In addition, such an influence of 8.0 kHz has been detected for the HHIE-S as well [20]. In contrast to the APHAB, the HHIE-S and the MAT are developed for screening use only. The APHAB is too large to play an important role in screening. Nevertheless, sensitivity and specificity are required characteristics for inventories in general [12].

At present, some rather difficult methods in conjunction with the APHAB to measure the quality of hearing aid fitting in patients with statutory insurance are used in Germany [3, 4]. They calculate relations of the differences of subscales to their means which can due to some problems in the result by arithmetic reasons. Of course, these methods are based on the difference of two APHAB forms, before and after hearing aid fitting. But going forward, it may be of benefit to patient and clinicians to instead use modified Eqs. (1) or (2a) for quality measurement of hearing aid fitting as well. However, further research is required to validate our results with these models. These models may be of particular benefit in cases in which the APHAB_u_ is being used as a screening inventory or as a primary audiological diagnostic method. Use of the logistic regression model to determine the diagnostic value of the APHAB_u_ may be justified by the weighted influence of the RV subscale. Recent investigations have found that the likelihood of individual compensatory effects is highest for BN and lowest for RV and that the influence of the EC subscale is limited to cases with increased hearing loss [7, 10]. In summary, our determination of the sensitivity and specificity of the APHAB_u_ adds to the knowledge of this widely used inventory in Germany. We suggest that future studies investigate the values of these parameters separately for each frequency. Together with the recently published percentile distribution curves and box plots of the unaided and aided APHAB and the resulting benefit [21] and together with the knowledge of mutual dependencies of APHAB_u_ scores, pure-tone thresholds, and speech-audiometric results, it is well possible to interpret an individual hearing loss.
